# Victims and Perpetrators of Child Sexual Abuse: Abusive Contact and Penetration Experiences

**DOI:** 10.3390/ijerph18189593

**Published:** 2021-09-12

**Authors:** Marta Ferragut, Margarita Ortiz-Tallo, Maria J. Blanca

**Affiliations:** 1 Faculty of Psychology, University of Málaga, 29071 Málaga, Spain; mortiz@uma.es (M.O.-T.); blamen@uma.es (M.J.B.); 2 Con.Ciencia Association, 29016 Málaga, Spain

**Keywords:** child sexual abuse, contact abuse, penetration, victims, perpetrators

## Abstract

Child sexual abuse (CSA) includes abusive contact experiences, which habitually impact the victim’s whole life. This study aims to analyze the characteristics of six CSA experiences with physical contact, including penetration, in a representative sample of the Spanish population. Participants were 1071 Spanish adults (53% males; M_age_: 45.37) who completed the Child Sexual Abuse Experiences Questionnaire. The victim’s age at the first episode, the perpetrator’s characteristics, and the number of times that each experience occurred were analyzed, taking into account gender differences. Results were reported for every experience independently. The most prevalent age at the first experience was from 6 years old onwards, but with differences in some experiences. The abuses usually happened more than once, committed by the same person. The most prevalent perpetrator is a male, although a female perpetrator is more prevalent in male victims. Most of the abuses were committed by an adult acquaintance, a strange adult, and other minors, with some gender differences. The implications of the results concerning every CSA experience are discussed, highlighting their value for future research and practice, the design of preventive programs, and early detection of CSA.

## 1. Introduction

Child sexual abuse (CSA) is a complex phenomenon referring to the involvement of a minor in acts that pursue the sexual gratification (physical or mental) of another person who is in a position of power or inequality, and in the absence of true consent, constituting an abusive and undesired experience [1]. CSA can include both non-contact and contact experiences [2]. In the research field, studies have considered diverse approaches to CSA, some of them analyzing CSA in a broad sense (including non-contact and contact abuse experiences), whereas others use a narrow definition (more restricted, such as forced intercourse or contact abuse) [3]. Contact abuse experiences include sexual acts, such as those involving penetration or intentional touching (fondling, rubbing, or kissing) without penetration [3,4,5]. It is widely known that these abuse experiences habitually impact the victims’ life, provoking important life-long physical, emotional, and social consequences [5,6,7,8].

Some meta-analyses examine CSA prevalence without differentiating between contact and non-contact experiences [9], whereas others differentiate three categories: non-contact, contact, and penetration experiences [10,11,12,13]. All meta-analyses found high variability in the prevalence rates and characteristics of CSA. Many factors may be influencing this heterogeneity. On the one hand, studies vary regarding the definitions of CSA (broad vs. narrow) and the experiences analyzed; on the other hand, methodological issues, such as different data collection procedures and sampling strategies, may yield different prevalence rates [12,14]. To gain more adequate CSA information, the analysis of every single abusive experience independently and the use of representative samples have been proposed [3,15].

Despite the variability, studies that analyze every abusive contact experience separately found that the most prevalent experience is fondling, followed by being forced to touch the abuser, and the least prevalent one is sexual intercourse with penetration [15,16,17,18,19]. Generally, it is found that most of the victims had experienced more than one kind of CSA [15,19,20]. Furthermore, it is consistently found that females report higher CSA prevalence than males [3,11,13,18,21,22]. However, some authors consider that this lower prevalence found in males may be explained by the fact that their experiences are not well represented by the questions used in the studies, males’ difficulties to identify their experiences as CSA—perhaps due to shame and social stereotypes (e.g., being judged as homosexual)—and also due to the low number of male participants in the studies [23,24,25,26,27,28].

Regarding the context of the abuse, the victim’s age and the relationship with the perpetrator have been postulated as relevant variables in the analysis of the CSA. Although some studies inform that all age groups below 18 are at risk of being sexually victimized [13], primary school age (between 6 and 12 years old) is the highest moment of risk for children to suffer CSA for the first time [15,16,20,29,30,31]. Empirical evidence has shown that most perpetrators of CSA were known to the victim, either family members or family friends and neighbors [13,19,32]. Intrafamilial abusers have been linked to more severe CSA than that perpetrated by acquaintances or strangers and are related to more contact experiences, earlier onset, longer duration, and more health consequences [15,20,29,33]. As the age of the victim increases, the profile of the perpetrator may change. Adolescence is a period of increased risk of CSA perpetrated by peers [11,30]. Differences in methodologies, classification, and types of CSA assessed may underlie the variability of the results regarding the relationship with the perpetrator [11]. Finally, it has been consistently reported that perpetrators are mostly males [33,34], although male victims report abuse by a woman more frequently than females [33,35].

In Spain, only two studies used a representative sample to study CSA prevalence [18,36]. Both studies found high rates of abuse experiences that include physical contact, and having suffered several experiences is habitual, with fondling as the most prevalent. López et al. [36] also found that the perpetrators were mostly men and equally distributed among known persons and strangers to the victim. The present study is a continuation of previous research [18] that determined the prevalence of different CSA experiences and disclosure variables. This recent study found, in contact CSA, that being fondled (16.9%) and rubbed (14.6%) were the experiences with higher prevalence, followed by being kissed (11.2%), having been asked to touch the abuser’s private parts (10.5%), and having had own private parts touched (10%). The lowest prevalence found was being forced to perform a sexual act involving penetration (2.8%). Ferragut et al. [18] also reported that women suffered significantly more contact abuse experiences, except for acts involving penetration, where similar rates in men and women were found.

Some of the previous studies with the Spanish population analyzed the relationship with the perpetrator as a function of the victim’s age and sex, in university samples or only in women [20,37]. These authors found that, before the age of 13, most aggressors tend to belong to the victim’s immediate environment, mainly friends or acquaintances in the case of males and relatives in the case of females [20,37]. In abuses starting after the age of 13, the number of unknown aggressors in females increases [20], as well as the number of peer aggressors [37].

In sum, meta-analysis research had reported high variability in the prevalence rates of CSA and inform that most of the studies analyze samples with a majority of women, university students, or clinical samples [3,9,12,22]. Authors suggest that studies analyzing every abusive experience independently and using representative samples are needed to acquire adequate knowledge of the CSA reality [3,15,20]. Contact experiences of CSA, the victim’s age when the abuse started, and the relationship with the perpetrator are important variables that may predict victims’ health consequences [38], but the heterogeneity of previous results requires a more detailed analysis of these variables in every CSA experience, differentiating between genders [15]. This study aims to analyze the characteristics of six different CSA experiences with physical contact, including penetration, in a representative Spanish sample, expanding the prevalence study carried out by Ferragut et al. [18] with unexplored variables. Specifically, we sought to contribute to a deeper understanding of the characteristics of CSA, taking into account gender differences, and analyzing the age of the victim at the first episode, the number of times that these experiences occurred, whether or not the perpetrator was the same person (in the case of more than one experience), and the type of perpetrator in each experience: gender and relationship with the victim. The findings of this study may serve as a basis for the design of social policies and the development of effective prevention programs that take into account the real characteristics of CSA.

## 2. Materials and Methods

### 2.1. Participants

A sample of 1071 Spanish adults (50.33% males) between 18 and 74 years old (M = 45.37, SD = 14.84) participated in this study. Concerning educational level, 33.6% of participants had finished university studies, 33.4% had elementary studies, 27.1% had completed high school, and 5.9% reported no schooling. Considering employment status, 48.5% of respondents were employed at that moment, 20.5% were unemployed, 16.7% were retired, 7.2% were homemakers, and 7.1% were students. Regarding marital status, 60.1% were married, 29.7% were single, 7.7% were divorced or separated, and 2.4% were widowed. This sample was representative of the general Spanish population in terms of gender, age, and region (north: 15.4%, center: 26.5%, south; 23%, and east: 35.1%), as recorded in the 2018 census report of Spain’s National Institute of Statistics, with a confidence level of 95% and a margin of error equal to 3%.

### 2.2. Instrument

#### Child Sexual Abuse Experiences Questionnaire

The Child Sexual Abuse Experience Questionnaire (CSAEQ), developed by Ferragut et al. [18] for a national prevalence study, was administered. This is an online questionnaire that retrospectively gathers the occurrence of different experiences during childhood. It includes demographic information and ten questions about experiences of CSA, including contact and non-contact experiences. For the present study, only the experiences with physical contact were analyzed. Respondents were asked about experiences that; (1) occurred when they were still legally a minor (younger than 18 years old), (2) involved an adult or another child who exceeded them in terms of age, development, strength, or authority, and (3) were felt to be inappropriate (i.e., not playing with a peer under equal conditions). For this study, six events were analyzed; participants had to answer yes or no if anyone ever: rubbed his/her private parts against them, fondled any part of the victim’s body, touched their private parts, ask the victim to touch the perpetrator’s private parts, kissed the victim, and forced the victim to perform a sexual act involving penetration. If participants responded yes to any of these experiences, they were then asked to indicate their age at the time of the first episode (younger than 6 years, 6–11 years, 12–15 years, and 16–17 years), how many times they had that experience (once, 2–3, 4–5, and more than 5 times), whether or not the perpetrator was the same person (in the case of more than one experience), the perpetrator’s gender (male, female, or both, in the case of more than one experience), and the relationship with the perpetrator. In this last question, participants had to answer yes or no to the following relationships: parent, adult family member, person responsible for childcare (teacher, instructor, babysitter, health staff, etc.), adult acquaintance, strange adult, and another minor.

### 2.3. Procedure

Data collection was performed by a specialist market research company, gathering responses through an online survey. This company employs a large number of people who receive incentives for completing surveys through points that can be used in an online store. All the participants signed a collaboration and data protection agreement. This company was awarded the ISO 26362 certification for access panels in marketing, opinion, and social research. The approximate time to complete this questionnaire was between 5 and 15 minutes. All participants were informed about the objectives and that the data would be treated anonymously for research purposes. They were requested to sign the informed consent and declare being over 18 years old to access the survey. The study was carried out following the Declaration of Helsinki and was approved by the Research Ethics Committee of the University of Malaga on 6 May 2020 (number 18-2020-H).

### 2.4. Data Analysis

For each type of CSA with contact, we analyzed the age at the first experience, the number of times that each CSA occurred, whether or not the perpetrator was the same person, the perpetrator’s gender, and the relationship between the victim and the perpetrator (parent, adult family member, person responsible for childcare (teacher, instructor, babysitter, health staff, etc.), adult acquaintance, strange adult, and another minor). We computed percentages and used the chi-square test and Fisher’s exact test to analyze differences by gender.

## 3. Results

We present the results of each CSA separately. Overall, of the 1071 participants, 298 (27.82%) had experienced some form of contact CSA.

### 3.1. Experience 1. “Somebody Rubbed His/Her Private Parts against Me”

Results of the rubbing experience are shown in Table 1. The most prevalent age at the first episode of this experience was between 6 and 11 years old (40.4%). Most of the victims (68.6%) declared that the experience happened twice or more. Of the latter, 57% stated that the abuse was performed by different people, mainly by a male. However, in comparison with female victims, a higher percentage of male victims reported that the rubbing was committed by a female perpetrator (18% vs. 3.8%). Regarding the relationship with the perpetrator, the highest percentages reported that the rubbing was performed by a strange adult, followed by an adult acquaintance, and another minor. The only gender difference involved a minor as the perpetrator: male victims declared a higher percentage (40.0%) of minor perpetrators than female victims (18.9%).

### 3.2. Experience 2. “Somebody Fondled Some Part of My Body”

Results of the fondling experience are shown in Table 2. The most prevalent age at the first episode of this type of abuse was in the range of 6–11 years old (38.7%). Most of the victims declared that the fondling happened more than once (63%), and 55.3% reported that the abuse was done by different perpetrators. Most of the victims also identified a male as the perpetrator, although a higher percentage of male victims than female victims reported that the fondling was committed by a female perpetrator (24.5% vs. 3%). Overall, the fondling was done mainly by an adult acquaintance, a strange adult, or another minor. However, females reported having been fondled by an adult family member at a higher rate than males (20.5% vs. 8.2%).

### 3.3. Experience 3. “Somebody Touched My Private Parts”

Results of the touching experience are shown in Table 3. The first episode of this experience was more prevalent in the age range of 6–11 years old (49.5%). The experience happened more than once for 57% of the victims, mainly with the same person. The touching was done mainly by a male, although female perpetrators were more frequent among male victims than among female ones (31.4% vs. 5.6%). Overall, this abuse was performed mainly by an adult acquaintance, a strange adult, or another minor. However, a higher percentage of female victims than male victims reported having been touched by an adult family member (23.6% vs. 8.6%).

### 3.4. Experience 4. “Somebody Asked Me to Touch His/Her Private Parts”

Results of the experience of touching the abuser are shown in Table 4. The most prevalent age at the first episode of this experience was in the range of 12–15 years old (33.9%). As in the previous cases, most of the victims (59.8%) reported having suffered this experience more than once, mainly by the same person. Likewise, most of the victims stated that the perpetrator was a male, although a higher percentage of male victims declared that the abuse was committed by a female perpetrator (29.5% vs. 12.5%). Overall, the request was made mainly by an adult acquaintance, another minor, and a strange adult.

### 3.5. Experience 5. “Somebody Kissed Me”

Results of the kissing experience are shown in Table 5. The most prevalent age at the first episode of this experience was in the range of 12–15 years old (39.2%). As in previous cases, most of the victims (63.3%) stated that they had had this experience more than once, mainly with different perpetrators. The most prevalent perpetrator was a male, although we found a higher percentage of male victims, in comparison with female victims, who declared that they had been kissed by a female perpetrator (66.7% vs. 6%). The kissing was performed mainly by another minor, an adult acquaintance, and a strange adult, and by another minor more frequently in male victims.

### 3.6. Experience 6. “Somebody Forced Me to Perform a Sexual Act Involving Penetration”

Results of the penetration experience are shown in Table 6. The first episode of this experience was more prevalent in the age range of 16–17 years old (46.7%), although there was quite a large percentage at the age of 6–11 (36.7%). More than half of the victims (56.7%) declared that this experience happened twice or more, mainly with the same person. Most of the victims stated that the perpetrator was a male, with no differences by gender. Regarding the relationship with the perpetrator, an adult acquaintance was the most prevalent, followed by a strange adult, an adult family member, and another minor. The difference between genders tended to be significant (*p* = 0.054) when a person responsible for childcare was the perpetrator, which was more prevalent for male victims than for female ones (25% vs. 0%).

**Figure 1:**
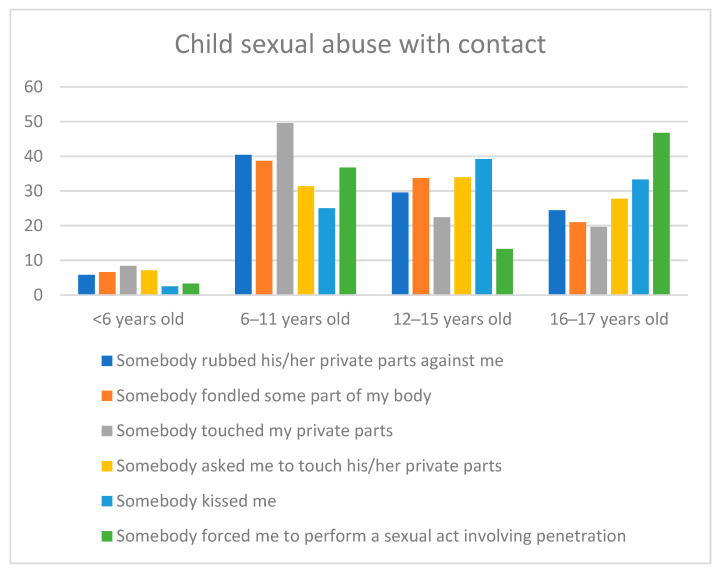
Age at the first episode as a function of the type of CSA with contact.

**Figure 2:**
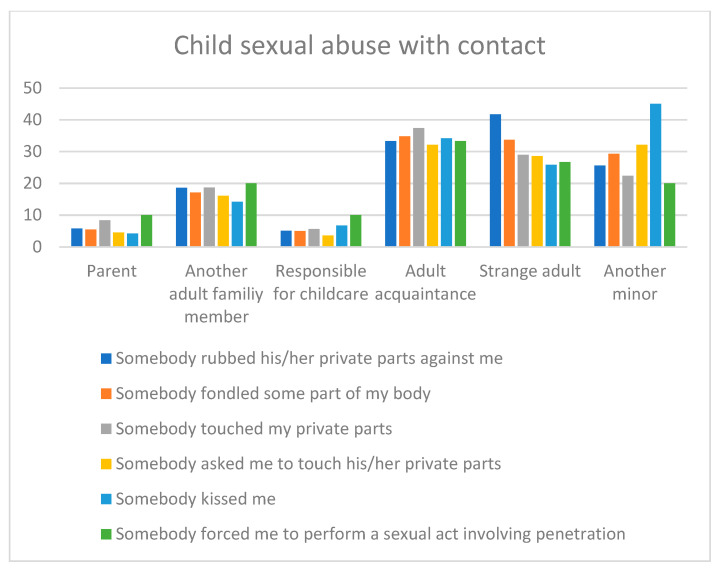
Perpetrator as a function of the type of CSA with contact.

In summary, regarding the six types of CSA with contact that were analyzed, we found that:

- There were no gender differences regarding the age at the first episode of contact CSA. The most prevalent age was from 6 years old onwards. The age comparison among the different types of CSA is shown in Figure 1.
- The abuses usually happened more than once, without gender differences.
- In cases where the abuses happened more than once, the most invasive abuses were committed by the same person (touching the victim’s private parts, asking the victim to touch the perpetrator’s private parts, and forcing the victim to perform a sexual act involving penetration). There were no gender differences.
- The most prevalent perpetrator was a male, although there were gender differences in most of the experiences, such that a female perpetrator was more prevalent in male victims.
- Most of the CSA experiences were committed by an adult acquaintance, a strange adult, and other minors. The comparison among the types of CSA is shown in Figure 2. However, there were some gender differences: males more frequently reported having been rubbed and kissed by another minor and tended to report having been forced to perform a sexual act involving penetration by a person responsible for childcare, whereas female victims more frequently reported having been fondled and touched by an adult family member.

## 4. Discussion

This study aimed to analyze the characteristics of six different CSA experiences with physical contact, including penetration, in a representative Spanish sample, expanding the prevalence study carried out by Ferragut et al. [18] with unexplored variables. Specifically, we sought to contribute to a deeper understanding of the characteristics of CSA, taking into account gender differences, and analyzing the victim’s age at the first episode, the number of times that these experiences occurred, whether or not the perpetrator was the same person (in the case of more than one experience), and the perpetrator profile in each experience: gender and relationship with the victim. The six types of CSA assessed include that someone: rubbed his/her private parts against the victim, fondled any part of the victim’s body, touched the victim’s private parts, asked the victim to touch the perpetrator’s private parts, kissed the victim, and forced the victim to perform a sexual act including penetration.

Regarding the age at the first episode, no gender differences were found in any of the experiences. The most prevalent general age to have suffered any CSA contact experience for the first time is from 6 years old onwards. The results confirmed that the age range between 6 and 11 years is a high-risk period, with increased prevalence for the first episode in three of the six experiences with contact assessed, in line with previous studies [15,16,20,29,30,31]. Some authors have informed that the earlier the abuse starts, the more severe it is and the more severe its consequences are [19,20]. This highlights the importance of the primary education stage for the prevention and early detection of CSA, implementing programs in the school context that protect children and help them identify and report the abuses. However, adolescence has also been found to be a period of high risk for CSA for both genders, with the age range from 12 to 15 years old being the most prevalent for two CSA experiences and with high percentages in the rest of the events. This result supports the findings of previous studies that consider adolescence as a moment of increased risk for sexual abuse and intercourse [11,21,30,39,40], and indicates the need to reinforce preventive programs in the adolescent stage.

Most of the abusive experiences had happened repeatedly (more than once), without gender differences. If the experience had happened more than once, the most invasive abuses were committed by the same person without gender differences (touching victims’ private parts, asking the victim to touch perpetrators’ private parts, and forcing the victim to perform a sexual act involving penetration). This result is consistent with previous research in Spain, which found that more than half of the contact abuses were continued over time [29]. Results suggest that victims of CSA with contact are very likely to suffer a chronic or repetitive situation with the same perpetrator when the abuses are related to touching intimate parts or sexual intercourse. It is shown that CSA is not usually an isolated experience, and the risk of revictimization is high both for girls and boys, which indicates the need for CSA prevention and detection at very early ages. Previous research has studied revictimization in CSA, finding that perceived parental care is the only protective factor against it [41]. This highlights the importance of involving parents in prevention programs, giving them the resources for early detection of sexual abuse, and teaching them how to provide support to their children.

Regarding the abuser characteristics, the most prevalent perpetrator is a male, although, in five of the six experiences of CSA, male victims more frequently reported having been abused by a female aggressor. This is in line with previous research that has consistently found that the perpetrators are males in a very high percentage [20,33,34] and with research that has also found that male victims report having been abused by a woman more frequently than female victims [33,35,42,43]. These results emphasize the importance of considering the female profile as a possible child sex offender and the male profile as a victim for future research and the design of prevention programs.

Concerning the relationship of the perpetrator with the victim, the results show that adults acquaintances, strange adults, and other minors are the three most common perpetrators in all the CSA experiences. This result may conflict with studies that indicate family members or caregivers as the most prevalent perpetrators [19,29], but it coincides with other studies stating that most perpetrators of contact abuse are extrafamilial: acquaintances or peers [15,33]. The relationship between the victim and the perpetrator has previously been related to the victim’s age, such that acquaintances are more probable in younger victims, whereas experiences with strangers and peers increase as the victim grows older [11,20,37]. In this study, we have computed each category of the relationship with the perpetrator separately for the analysis, but when adding all the “known adult perpetrators” (parent, another adult family member, person responsible for childcare, and adult acquaintance), the percentage is always higher than that of strange adults in all the experiences, as found in previous research [13,19,32]. This result highlights that, although the relationship with the perpetrator may vary depending on the type of CSA experience, the highest risk is usually found in the child’s close social environment.

There were some gender differences in the relationship with the perpetrator: males reported a higher rate of having been rubbed and kissed by another minor and tended to report having been forced to perform a sexual act involving penetration by a person responsible for childcare, whereas female victims reported a higher rate of having been fondled and touched by an adult family member. Pereda and Forns [37] also found gender differences, with friends or acquaintances being more common perpetrators in the case of males and family members in the case of females. This result helps to provide knowledge for detection and prevention programs, highlighting a possible differential pattern of male and female victims in the relationship with the perpetrator, depending on the type of experience. Efforts in prevention and detection could be oriented toward experiences with other minors and persons responsible for childcare in boys and toward experiences in the family context in girls.

The results about the peers as perpetrators, especially in male victims, deserve attention. Previous studies stated that when peer abuse is included in the analysis, higher rates of CSA are found, especially in males [11,21]. This result has also been reported in previous studies in Spain [36,37]. Future research should address sexual abuse among peers, especially in males.

Finally, the experience that includes sexual acts with penetration deserves a specific section, given that this type of CSA is associated with severe mental and physical health consequences [43,44], including sexually transmitted infections or unwanted pregnancies [45]. Ferragut et al. [18] found a prevalence of 2.8%, indicating that approximately 1 in 35 minors had been abused with penetration in Spain. The results found in the present study show that this CSA has a similar pattern for male and female victims regarding the victim’s age at the first episode, number of times that it happened, and the perpetrator’s gender. Overall, the most prevalent age at the first episode was between 16–17 years (46.7%), followed by 6–11 years (36.7%); more than half of the victims reported that the experience occurred more than once (56.7%), with the same person (58.8%), mainly with a male perpetrator (86.7%) who was an adult acquaintance (33.3%). These findings are consistent with those reported by Mohler-Kuo et al. [33], who found that CSA with penetration occurs mainly during adolescence and that most perpetrators were known to the victim. As mentioned above, the gender difference tended to be significant, *p* = 0.054, when a person responsible for childcare was the perpetrator and was more prevalent for male victims. It should be noted that 25% of the males abused with penetration reported being abused by a person responsible for childcare compared to 0% of the females. A person responsible for childcare is in a position of authority over the victims, and perpetrators can use this advantage to commit an abuse, and this has been found to be related to the risk of revictimization [46].

The results of this study may serve to alert the authorities and the adults responsible for childcare to direct their efforts to create safe spaces for children. The school and the responsible adults must be references of safety and care, creating an environment in which the risk of aggression against children is detected and minimized. To combat sexual abuse, the communities, public institutions, and society must participate in an informed discourse about CSA [47]. For this purpose, the implementation of education programs, including sexual abuse information and resources as part of the school and teacher education curricula, as well as support services to students in the school context, could be an optimal way to detect and prevent abuse and should be considered mandatory [48].

This study has some limitations that must be acknowledged. This is a cross-sectional study that analyzes childhood experiences reported by adults retrospectively, so it is conditioned by the adults’ memories and possibly affected by recall bias. Further, the fact that participants were informed about the purpose of the study may have introduced an element of self-selection. However, it also has a number of strengths. It analyzes the characteristics of the different contact abuse experiences independently, so the results can be itemized, allowing comparisons with other studies. Furthermore, the sample included is representative of the Spanish population, covering all age ranges and with comparable proportions of men and women, which allows comparison between genders.

## 5. Conclusions

The findings of this study show that every CSA experience has its own characteristics and context. However, it can be assumed that primary education (from 6 years old) is generally one of the highest moments of risk for suffering the first abusive contact experience. Therefore, this is a key moment to start prevention and detection programs of CSA. However, these programs should be reinforced in the adolescent stage, where important sexual risks may occur, such as sexually abusive intercourse with penetration.

Males and females do not differ significantly in some of the CSA patterns, and they usually suffer these abuses at a similar age of onset and repeatedly by the same perpetrator. They are commonly perpetrated by an extrafamilial male, who may be an acquaintance, a stranger, or a peer. However, males are at a higher risk of suffering CSA experiences with a female perpetrator. Males more frequently experienced being rubbed and kissed by another minor and being forced to perform a sexual act involving penetration by a person responsible for childcare, whereas females more frequently reported having been fondled and touched by an adult family member.

This information could be useful for future research and practice, as well as for the design of preventive programs and the early detection of CSA. Preventive programs should also target boys to make them understand that they can also be abused by a woman or a peer, preventing them from feeling ashamed of the situation and encouraging them to ask for help. Overall, efforts by the school, the family, and social policies should be combined to minimize the risk of suffering CSA.

## Figures and Tables

**Table 1:** Percentage of victim’s age at the first experience and perpetrator’s characteristics as a function of gender for “somebody rubbed his/her private parts against me”.

Variables	*N*	%	Male (*n* = 50)	Female (*n* = 106)	χ^2^
Age at first experience					
<6	9	5.8	4.0	6.6	0.46
6–11	63	40.4	42.0	39.6	
12–15	46	29.5	30.0	29.2	
16–17	38	24.4	24.0	24.5	
Number of times					
Once	49	31.4	42.0	26.4	5.95
2–3	65	41.7	42.0	41.5	
4–5	14	9.0	6.8	10.4	
>5	28	17.9	10.0	21.7	
Perpetrator (more than one experience)					
Same person	46	43.0	44.8	42.3	0.06
Different people	61	57.0	55.2	57.7	
Gender of perpetrator					
Male	140	89.7	80.0	94.3	9.03 ^a,^**
Female	13	8.3	18.0	3.8	
Both	3	1.9	2.0	1.9	
Relationship with perpetrator					
Parent	9	5.8	2.0	7.5	1.92
Another adult family member	29	18.6	12.0	21.7	2.11
Responsible for childcare	8	5.1	6.0	4.7	0.11
Adult acquaintance	52	33.3	36.0	32.1	0.24
Strange adult	65	41.7	32.0	46.2	2.83
Another minor	40	25.6	40.0	18.9	7.96 **

Note. ^a^ Bootstrapping. ** *p* < 0.01.

**Table 2:** Percentage of victims’ age at the first experience and perpetrator’s characteristics as a function of gender for “somebody fondled some part of my body”.

Variables	*N*	%	Male (*n* = 49)	Female (*n* = 132)	χ^2^
Age at first experience					
<6	12	6.6	6.1	6.8	2.33
6–11	70	38.7	34.7	40.2	
12–15	61	33.7	30.6	34.8	
16–17	38	21.0	28.6	18.2	
Number of times					
Once	67	37.0	44.9	34.1	3.03
2–3	65	35.9	36.7	35.6	
4–5	17	9.4	6.1	10.6	
>5	32	17.7	12.2	19.7	
Perpetrator (more than one experience)					
Same person	51	44.7	48.1	43.7	0.17
Different people	63	55.3	51.9	56.3	
Gender of perpetrator					
Male	162	89.5	73.5	95.5	20.61 ^a,^**
Female	16	8.8	24.5	3.0	
Both	3	1.7	2.0	1.5	
Relationship with perpetrator					
Parent	10	5.5	2.0	6.8	1.56
Another adult family member	31	17.1	8.2	20.5	3.80 *
Responsible for childcare	9	5.0	6.1	4.5	0.19
Adult acquaintance	63	34.8	30.6	36.4	0.52
Strange adult	61	33.7	36.7	32.6	0.28
Another minor	53	29.3	30.6	28.8	0.06

Note. ^a^ Bootstrapping. ** *p* < 0.01. * *p* < 0.05.

**Table 3:** Percentage of victims’ age at the first experience and perpetrator’s characteristics as a function of gender for “somebody touched my private parts”.

Variables	*N*	%	Male (*n* = 35)	Female (*n* = 72)	χ^2^
Age at first experience					
<6	9	8.4	8.6	8.3	3.47
6–11	53	49.5	37.1	55.6	
12–15	24	22.4	28.6	19.4	
16–17	21	19.6	25.7	16.7	
Number of times					
Once	46	43.0	45.7	41.7	5.75
2–3	33	30.8	40.0	26.4	
4–5	9	8.4	8.6	8.3	
>5	19	17.8	5.7	23.6	
Perpetrator (more than one experience)					
Same person	39	63.9	73.7	59.5	1.14
Different people	22	36.1	26.3	40.5	
Gender of perpetrator					
Male	89	83.2	62.9	93.1	15.40 ^a,^**
Female	15	14.0	31.4	5.6	
Both	3	2.8	5.7	1.4	
Relationship with perpetrator					
Parent	9	8.4	5.7	9.7	0.50
Another adult family member	20	18.7	8.6	23.6	3.51 *
Responsible for childcare	6	5.6	8.6	4.2	0.86
Adult acquaintance	40	37.4	34.3	38.9	0.21
Strange adult	31	29.0	31.4	27.8	0.15
Another minor	24	22.4	25.7	20.8	0.32

Note. ^a^ Bootstrapping. ** *p* < 0.01. * *p* < 0.05.

**Table 4:** Percentage of victims’ age at the first experience and perpetrator’s characteristics as a function of gender for “somebody asked me to touch his/her private parts”.

Variables	*N*	%	Male (*n* = 44)	Female (*n* = 68)	χ^2^
Age at first experience					
<6	8	7.1	9.1	5.9	2.79 ^a^
6–11	35	31.3	36.4	27.9	
12–15	38	33.9	25.0	39.7	
16–17	31	27.7	29.5	26.5	
Number of times					
Once	45	40.2	50.0	33.8	4.89
2–3	42	37.5	34.1	39.7	
4–5	9	8.0	9.1	7.4	
>5	16	14.3	6.8	19.1	
Perpetrator (more than one experience)					
Same person	41	61.2	72.7	55.6	1.85
Different people	26	38.8	27.3	44.4	
Gender of perpetrator					
Male	98	87.5	70.5	98.5	19.25 **
Female	14	12.5	29.5	1.5	
Both	0.0	-	-	-	
Relationship with perpetrator					
Parent	5	4.5	0.0	7.4	3.38
Another adult family member	18	16.1	11.4	19.1	1.20
Responsible for childcare	4	3.6	6.8	1.5	2.21
Adult acquaintance	36	32.1	31.8	32.4	0.01
Strange adult	32	28.6	22.7	32.4	1.21
Another minor	36	32.1	36.4	29.4	0.59

Note. ^a^ Bootstrapping. ** *p* < 0.01.

**Table 5:** Percentage of victims’ age at the first experience and perpetrator’s characteristics as a function of gender for “somebody kissed me”.

Variables	*N*	%	Male (*n* = 36)	Female (*n* = 84)	χ^2^
Age at first experience					
<6	3	2.5	5.6	1.2	2.56 ^a^
6–11	30	25.0	27.8	23.8	
12–15	47	39.2	38.9	39.3	
16–17	40	33.3	27.8	35.7	
Number of times					
Once	44	36.7	36.1	36.9	1.29 ^a^
2–3	54	45.0	50.0	42.9	
4–5	6	5.0	5.6	4.8	
>5	16	13.3	8.3	15.5	
Perpetrator (more than one experience)					
Same person	32	42.1	43.5	41.5	0.03
Different people	44	57.9	56.5	58.5	
Gender of perpetrator					
Male	85	70.8	25.0	90.5	54.83 ^a,^**
Female	29	24.2	66.7	6.0	
Both	6	5.0	8.3	3.6	
Relationship with perpetrator					
Parent	5	4.2	0.0	6.0	2.24
Another adult family member	17	14.2	11.1	15.5	0.40
Responsible for childcare	8	6.7	5.6	7.1	0.10
Adult acquaintance	41	34.2	25.0	38.1	1.92
Strange adult	31	25.8	22.2	27.4	0.35
Another minor	54	45.0	61.1	38.1	5.40 *

Note. ^a^ Bootstrapping. ** *p* < 0.01. * *p* < 0.05.

**Table 6:** Percentage of victims’ age at the first experience and perpetrator’s characteristics as a function of gender for “somebody forced me to perform a sexual act involving penetration”.

Variables	*N*	%	Male (*n* = 12)	Female (*n* = 18)	χ^2^
Age at first experience					
<6	1	3.3	0.0	5.6	1.22 ^a^
6–11	11	36.7	41.7	33.3	
12–15	4	13.3	8.3	16.7	
16–17	14	46.7	50.0	44.4	
Number of times					
Once	13	43.3	33.3	50.0	0.85 ^a^
2–3	11	36.7	41.7	33.3	
4–5	2	6.7	8.3	5.6	
>5	4	13.3	16.7	11.1	
Perpetrator (more than one experience)					
Same person	10	58.8	50.0	66.7	0.49
Different people	7	41.2	50.0	33.3	
Gender of perpetrator					
Male	26	86.7	75.0	94.4	2.36
Female	4	13.3	25.0	5.6	
Both	0	-	-	-	
Relationship with perpetrator					
Parent	3	10.0	8.3	11.1	0.06
Another adult family member	6	20.0	16.7	22.2	0.14
Responsible for childcare	3	10.0	25.0	0.0	5.0 *
Adult acquaintance	10	33.3	50.0	22.2	2.5
Strange adult	8	26.7	33.3	22.2	0.45
Another minor	6	20.0	8.3	27.8	1.71

Note. ^a^ Bootstrapping. * *p* < 0.05.

## Data Availability

The data presented in this study are available on request from the corresponding author.
